# Changes in Biologically Active Compounds in *Pinus sylvestris* Needles after *Lymantria monacha* Outbreaks and Treatment with Foray 76B

**DOI:** 10.3390/plants13020328

**Published:** 2024-01-22

**Authors:** Vytautas Čėsna, Ieva Čėsnienė, Vaida Sirgedaitė-Šėžienė, Diana Marčiulynienė

**Affiliations:** Institute of Forestry, Lithuanian Research Centre for Agriculture and Forestry, Liepų 1, Girionys, LT-53101 Kaunas, Lithuania; ieva.cesniene@lammc.lt (I.Č.); vaida.seziene@lammc.lt (V.S.-Š.); diana.marciulyniene@lammc.lt (D.M.)

**Keywords:** *Bacillus thuringiensis*, chlorophylls, MDA, nun moth, Scots pine, secondary metabolites

## Abstract

Due to climate warming, the occurrence of *Lymantria monacha* outbreaks is predicted to become more frequent, causing repeated and severe damage to conifer trees. Currently, the most effective way to control the outbreaks is aerial spraying with the bioinsecticide Foray 76B. The present study aimed to determine the impact of both: (i) *L. monacha* outbreaks and (ii) treatment with Foray 76B on tree resistance through the synthesis of polyphenols (TPC), flavonoids (TFC), photosynthetic pigments (chlorophyll a and b, carotenoids), lipid peroxidation (MDA), and soluble sugars (TSS) in *Pinus sylvestris* needles. Samples were collected from visually healthy (control), damaged/untreated, and damaged/Foray 76B-treated plots in 2020 and 2021 (following year after the outbreaks). The results revealed that *L. monacha* outbreaks contributed to the increase in TPC by 34.1% in 2020 and 26.7% in 2021. TFC negatively correlated with TPC, resulting in 17.6% and 11.1% lower concentrations in *L. monacha*-damaged plots in 2020 and 2021, respectively. A decrease in MDA was found in the damaged plots in both 2020 and 2021 (10.2% and 23.3%, respectively), which was associated with the increased synthesis of photosynthetic pigments in 2021. The research results also showed that in the following year after the outbreaks, the increase in the synthesis of photosynthetic pigments was also affected by the treatment with Foray 76B. Moreover, the increase in the synthesis of TPC and photosynthetic pigments in the damaged plots in 2021 illustrates the ability of pines to keep an activated defense system to fight biotic stress. Meanwhile, a higher synthesis of photosynthetic pigments in Foray 76B-treated plots indicates a possible effect of the treatment on faster tree growth and forest recovery after *L. monacha* outbreaks.

## 1. Introduction

In recent decades, coniferous forests have been affected by various biotic and abiotic stressors, the frequency of which has increased due to a warming climate [1,2]. This increases tree mortality not only directly through drought and heat stress [3,4] but also indirectly by increasing their susceptibility to pests and pathogens [5]. Simultaneously, accelerating warming is promoting some insect development and outbreaks due to earlier food supplies and longer growing seasons [6]. These include Lepidoptera (moths and butterflies), which are commonly known as one of the most important groups of herbivores and pollinators, containing a huge vast prey biomass and hosting an extensive specialized web of parasitic Hymenoptera, and playing an important role in the functioning of our ecosystems [7]. However, some Lepidoptera are significant economic pests whose occurrence is increasing due to favorable overwintering conditions influenced by a warming climate [8], which poses a significant threat to the coniferous forests in Central, Eastern, and Northern Europe [9,10].

In Lithuania, *Lymantria monacha* L. (Lepidoptera: Lymantriidae) is among the main defoliators of Scots pine forests, whose population reaches outbreak level every 7–11 years, with the trends occurring more frequently in the last decades [9]. Pest outbreaks have been observed since the 18th century [11], with the latest outbreak in 2018–2020 where more than 3500 ha of pine stands were damaged [12]. The outbreaks of *L. monacha* also occur periodically in other Central and Northern European countries, including Poland [13], the Czech Republic [14], Germany [9], and Latvia [6]. The expansion of *L. monacha* infestations is marked by high population densities of the larvae [15], which feed on coniferous tree needles, removing the photosynthetic tissue critical for plant maintenance and growth [16]. It causes widespread damage to forest resources including increment loss, dieback, and tree mortality [10]. To prevent this and minimize the damage caused by insects, conifers have anatomical, mechanical, biochemical, and molecular defense mechanisms [9]. These involve a complex mixture of constitutive (preformed) and inducible (acquired) defenses that must be considered when developing forest management strategies to control tree diseases [17].

Among plant defensive traits, chemical barriers, such as a variety of plant secondary metabolites (PSMs), including flavonoids, which represent a rich group of polyphenols, are essential compounds in the ability of plants to interact with and adapt to the environment [18]. Previous research has noted that insect herbivory might disturb the biosynthesis of PSMs and change photosynthesis activity in plant tissue [19,20]. For instance, insect defoliators partially destroy the photosynthetic apparatus resulting in excess energy storage and elevated concentration of reactive oxygen species (ROS) by causing photo-oxidative stress and activating photoprotective and antioxidative defense systems [21]. In response to oxidative stress plants undergo a process known as lipid peroxidation, which leads to alterations in the protein and nucleic acid composition of the cell membrane system [20,22,23,24,25]. Malondialdehyde (MDA) is a substance produced by membrane lipids whose higher levels in plant tissues signify a higher degree of damage [22].

In addition, the process of photosynthesis is not entirely synchronized with the demands of carbon sinks, thereby indicating that trees possess the ability to accumulate non-structural carbohydrates (NSC), for instance, soluble sugars and starch within their tissues [26]. Sugars as key signaling and balancing molecules have been shown to modulate stress response [27], growth and development [28], and PSMs synthesis [29], and it is implicated in indirect antioxidant mechanisms against ROS [30,31]. Sugars can protect plant cells against the impact of ROS by acting as both compatible solutes and direct scavengers [31]. They also serve as the primary source of energy and carbon skeletons for producing the metabolites essential for both enzymatic and non-enzymatic antioxidant responses [30].

In most cases, insect mass outbreaks do not kill the host tree; however, the outbreaks weaken the defense mechanisms of trees, inducing further attacks by other pests and pathogens [32]. Therefore, to control the massive expansion of insect pests, the application of additional measures is required [33]. In the case of the nun moth outbreaks, aerial application of the bioinsecticide Foray 76B [34], which is made from the soil bacterium *Bacillus thuringiensis* subspecies *kurstaki* Strain ABTS-351 (Btk), which forms spores with Cry or Cyt proteins inside the defoliator during its growth cycle [34,35], is needed. Even though Btk is considered toxic to targeted lepidopterans, and numerous impact studies show this bacterium’s safety on non-target organisms, there is a gap in knowledge regarding the effect of Btk on the accumulation of defensive compounds in conifer needles. Also, the information about the effect of various biotic stressors on *P. sylvestris* defense apparatus is limited [36]. Therefore, the present study aimed to investigate the impact of *L. monacha* outbreaks and the treatment with Foray 76B on the accumulation of PSMs, photosynthetic pigments, lipid peroxidation, and sugars in *P. sylvestris* needles. The findings of this study can be helpful for a better understanding of complex plant-insect interactions, particularly how pines respond to *L. monacha* damage and Foray 76B treatment through their defense mechanisms. Such knowledge provides significant insights into tree resistance and stand recovery after *L. monacha* outbreaks and can contribute to the development of sustainable forest conservation strategies that support ecosystem functions.

## 2. Results

### 2.1. Effect of L. monacha Outbreaks on Total Polyphenol (TPC) and Total Flavonoid (TFC) Content

**Figure 1:**
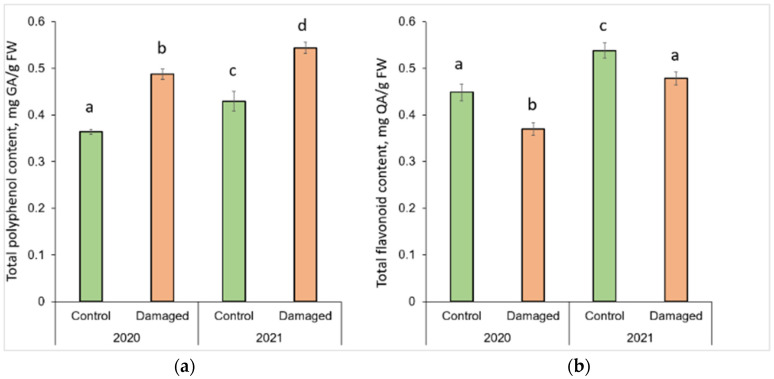
Graphical representation of (**a**) total polyphenol (TPC) and (**b**) total flavonoid (TFC) content in the needle of *P. sylvestris* samples from control (undamaged) and *L. monacha* damaged plots in 2020 and 2021. Mean values ± SE followed by the same lower-case letters indicate non-significant differences (*p* > 0.05) between different groups and years.

Graphical representation of column charts revealed changes in the concentration of secondary metabolites in *P. sylvestris* needles in *L. monacha* damaged and undamaged (control group) plots in both 2020 (outbreak period) and 2021 (following year after the outbreaks) (Figure 1). TPC significantly increased (*p* < 0.05) by 34.1% (in 2020) and 26.7% (in 2021) in the needle samples from damaged plots, compared to the control group (*p* < 0.05) (Figure 1a). However, in the needles from both damaged (by 11.5%) and control (by 18.0%) plots, TPC was higher in 2021 than in 2020. In contrast to TPC, TFC in the needle of *P. sylvestris* samples from damaged plots decreased by 17.6% in 2020 and 11.1% in 2021, compared to the control group (Figure 1b). Moreover, in 2021 TFC was higher in the needles from both damaged (by 29.4%) and control (by 19.9%) plots, than in 2020.

### 2.2. Effect of L. monacha Outbreaks on Chlorophyll a (Chl a), Chlorophyll b (Chl b), and Carotenoids (Caro)

**Figure 2:**
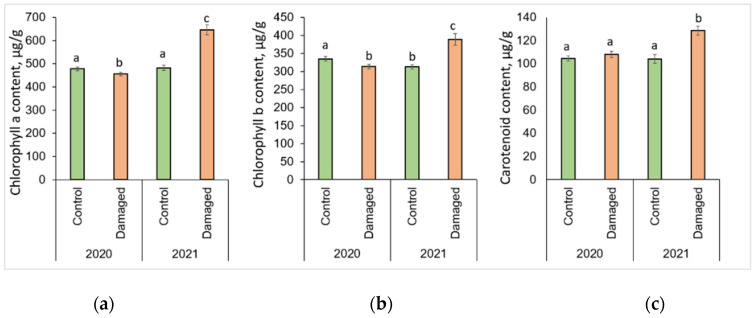
Graphical representation of (**a**) chlorophyll a (Chl a); (**b**) chlorophyll b (Chl b); (**c**) carotenoid (Caro) content in the needle of *P. sylvestris* samples from control (undamaged) and *L. monacha* damaged plots in 2020 and 2021. Mean values ± SE followed by the same lowercase letters indicate non-significant differences (*p* > 0.05) between different groups and years.

The changes in photosynthesis pigments after *L. monacha* outbreaks are shown in Figure 2. *P. sylvestris* needles collected in damaged plots in 2020 were determined by a significantly lower (*p* < 0.05) concentration of Chl a (by 4.7%) and Chl b (by 6.4%), compared to the needles of the control group (Figure 2a,b). In the following year (2021), the concentrations of Chl a, Chl b, and Caro were higher in the needle samples from damaged than from control plots, by 33.8%, 24.6%, and 23.5%, respectively (Figure 2a–c). The concentrations of all assessed photosynthetic pigments in the needles from damaged plots in 2021 were higher than in 2020, by 41.7% (Chl a), 24.3% (Chl b), and 19.0% (Caro), respectively.

### 2.3. Effect of L. monacha Outbreaks on Lipid Peroxidation (MDA) and Total Soluble Sugars (TSS)

**Figure 3:**
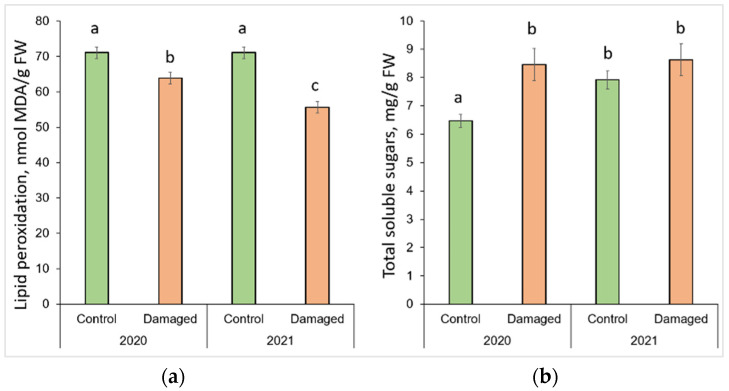
Graphical representation of (**a**) lipid peroxidation (MDA); (**b**) total soluble sugar (TSS) content in the needle of *P. sylvestris* samples from control (undamaged) and *L. monacha* damaged plots in 2020 and 2021. Mean values ± SE followed by the same lower-case letters indicate non-significant differences (*p* > 0.05) between different groups and years.

The changes in lipid peroxidation and total soluble sugars after *L. monacha* outbreaks are shown in Figure 3. The obtained results revealed that the level of MDA decreased significantly (*p* < 0.05) by 10.2% in 2020, and 23.3% in 2021 in the needle samples from damaged plots, compared to the control group (*p* < 0.05) (Figure 3a). It was determined that in 2021, the level of MDA in the needles from damaged plots decreased by 12.8%, compared to 2020. The results also showed that in 2020, the concentration of TSS in the needle samples from damaged plots increased by 30.9%, compared to the control group (Figure 3b). Meanwhile, in 2021, the concentration of TSS in the needles of the control group increased by 22.5%, compared to 2020.

### 2.4. Impact of the Treatment with Foray 76B on Total Polyphenol (TPC) and Total Flavonoid (TFC) Content

**Figure 4:**
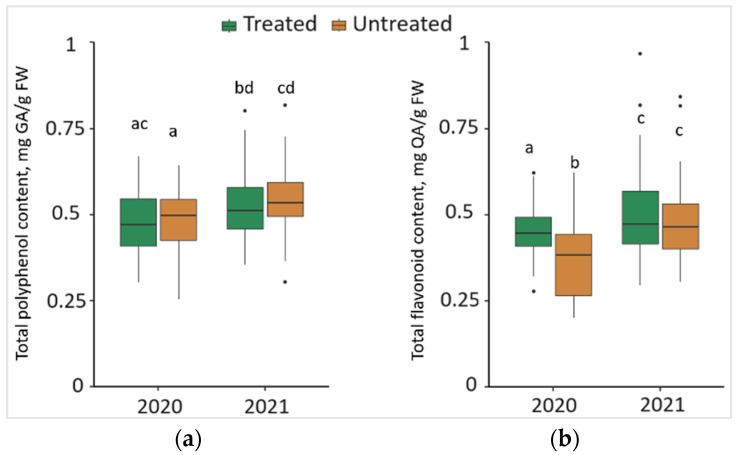
The boxplots and the outliers (black dots) of (**a**) total polyphenol (TPC); and (**b**) total flavonoid (TFC) content in the needles of *P. sylvestris* samples from Foray 76B treated and untreated plots in 2020 and 2021. Mean values ± SE followed by the same lower-case letters indicate non-significant differences (*p* > 0.05) between different groups and years.

Comparing the changes from different years, it was observed that TPC in the needle samples significantly increased (*p* < 0.05) by 8.3% in treated and 11.5% in untreated plots, with 0.04 mg/g higher median values both in treated and untreated plots in 2021 (Figure 4a). Meanwhile, in 2020, TFC in the needles from treated plots significantly increased by 22.5%, compared to the needle samples from untreated plots, with a 0.07 mg/g higher median value (Figure 4b). Comparing the changes from different years, it was found that TFC in the needle samples was significantly higher by 9.8% in treated and 29.4% in untreated plots in 2021, with higher median values (by 0.03 mg/g and 0.08 mg/g, respectively).

### 2.5. Impact of the Treatment with Foray 76B on the Content of Chlorophyll a (Chl a), Chlorophyll b (Chl b), and Carotenoids (Caro)

**Figure 5:**
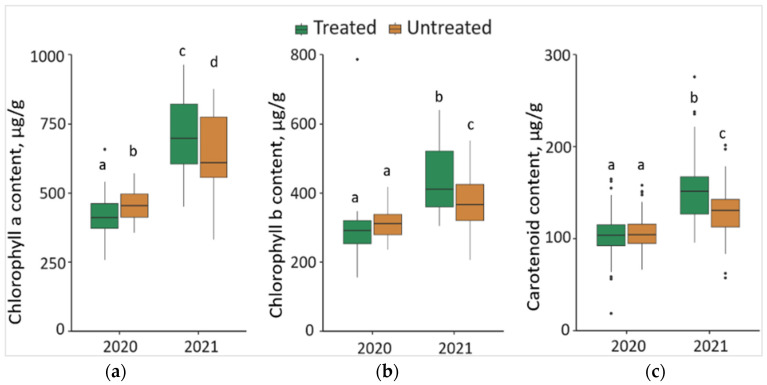
The boxplots and the outliers (black dots) of (**a**) chlorophyll a (Chl a); (**b**) chlorophyll b (Chl b); (**c**) carotenoid (Caro) contents in the needles of *P. sylvestris* samples from Foray 76B treated and untreated plots in 2020 and 2021. Mean values ± SE followed by the same lower-case letters indicate non-significant differences (*p* > 0.05) between different groups and years.

The synthesis of Chl a in 2020 was significantly lower (*p* < 0.05) by 7.7% in the needles from treated than from untreated plots, with a 42.8 μg/g lower median value (Figure 5a). In contrast, in 2021, the synthesis of Chl a in the needle samples from treated plots was significantly higher by 11.8%, compared to the needle samples from untreated plots, with a 93.17 μg/g higher median value. Comparing the changes between different years, it was found that the concentration of Chl a in the needle samples significantly increased by 71.5% in treated and 41.7% in untreated plots in 2021, with respective higher median values of 293.05 μg/g and 157.09 μg/g.

The concentration of Chl b in 2021 was significantly higher (*p* < 0.05) by 12.5% in the needle samples from treated than from untreated plots, with a 42.97 μg/g higher median value (Figure 5b). Comparing the changes between different years, it was noted that the concentration of Chl b in the needle samples significantly increased by 50.0% in treated and 24.3% in untreated plots in 2021, with higher median values (by 119.10 μg/g and 56.78 μg/g, respectively).

In 2021, the concentration of Caro was significantly higher (*p* < 0.05) by 17.7% in the needles from treated than untreated plots, with a higher 21.15 μg/g median value (Figure 5c). Comparing the changes between 2020 and 2021, it was found that the concentration of Caro in the needles of *P. sylvestris* samples significantly increased by 45.5% in treated and 19.0 in untreated plots in 2021, with, respectively 47.98 μg/g and 26.10 μg/g higher median values.

### 2.6. Impact of the Treatment of Foray 76B on Lipid Peroxidation (MDA) and Total Soluble Sugars (TSS)

**Figure 6:**
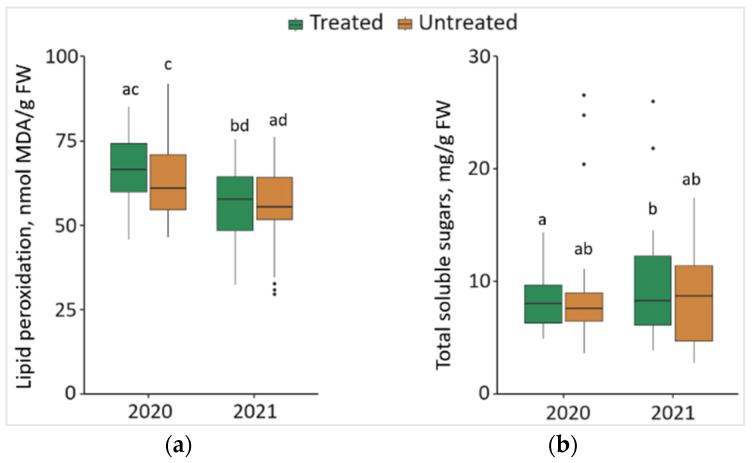
The boxplots and the outliers (black dots) of (**a**) lipid peroxidation (MDA); and (**b**) total soluble sugars (TSS) content in the *P. sylvestris* needles from Foray 76B treated and untreated plots in 2020 and 2021. Mean values ± SE followed by the same lower-case letters indicate non-significant differences (*p* > 0.05) between different groups and years.

Changes in MDA and TSS of the *P. sylvestris* needles from treated and untreated plots are shown in Figure 6. Comparing the changes between different years, it was noted that the level of MDA in the needle samples significantly decreased (*p* < 0.05) by 16.0% in treated and 12.8% in untreated plots in 2021, with lower median values (by 8.68 nmol/g and by 5.53 nmol/g, respectively) (Figure 6a). The results showed that the concentration of TSS was significantly higher by 14.2% in the needle samples from treated plots in 2021 than in 2020, with 0.23 mg/g higher median values (Figure 6b).

### 2.7. Correlation and Principal Component Analysis (PCA) between Biochemical Compounds

**Figure 7:**
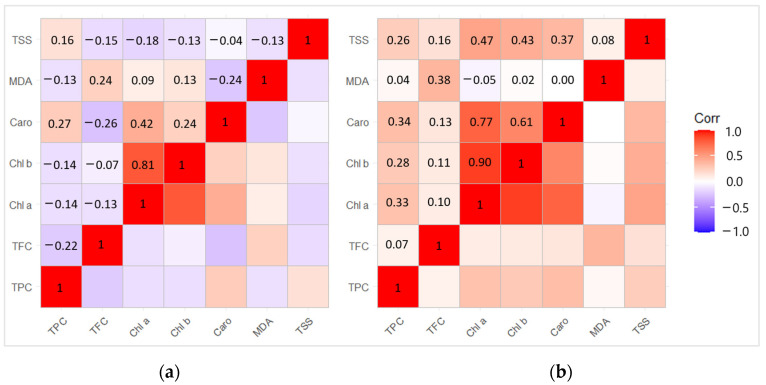
Matrix of correlation in (**a**) the year of *L. monacha* outbreaks (2020); (**b**) the following year after outbreaks (2021) between different biological active compounds based on the Pearson correlation coefficient.

The correlation analysis revealed positive dependence between Chl b − Chl a (r = 0.81) and Caro − Chl a (r = 0.42) (*p* < 0.05) in 2020 (Figure 7a). Meanwhile, the correlation between the biologically active compounds in 2021 showed a strong positive dependence between Chl b − Chl a (r = 0.90), Caro − Chl a (r = 0.77), TSS − Chl a (r = 0.47), Caro − Chl b (r = 0.61), and TSS − Chl b (r = 0.43) (*p* < 0.05) (Figure 7b).

**Figure 8:**
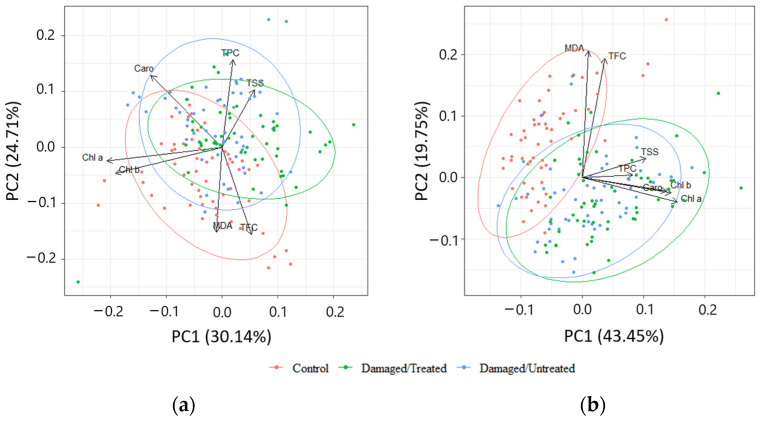
Principal component analysis (PCA) displaying the first and second components (PCs) from the data of (**a**) 2020; (**b**) 2021. The colors represent different groups: red—control; green—damaged/treated; blue—damaged/untreated samples. The loading vectors show the correlation between different biologically active compounds.

PCA of biologically active compounds displayed high overlapping between the needle samples from control, damaged/treated, and damaged/untreated plots in 2020 (Figure 8a). High overlapping between the needle samples from damaged/treated and damaged/untreated plots was also determined in 2021, while the control samples were less overlapped (Figure 8b). The first four of the seven principal components explained 84.9% of the total variance of the parameters, while the first two—63.2% in 2020 (Table 1). The first four of the seven principal components explained 80.4% of the total variance of the parameters, while the first two—54.8% in 2021. MDA and TFC were negatively linked to PC2 in 2020, while Chl a and Chl b were grouped together and were negatively linked to PC1 (Table 2). TPC and TSS were positively linked to PC2 in 2020. TPC and TSS as well as Chl a, Chl b, and Caro were grouped together and all these biologically active compounds were positively linked to PC1 in 2021.

## 3. Discussion

It is well known that insect mass outbreaks can disrupt the principles of sustainable forest ecosystems, making their recovery a difficult and prolonged process [37]. One of the most important defense systems of plants against such biotic stressors is their active mechanisms to produce chemical compounds that can repel dangerous insects or attract their enemies [38,39]. Previous studies were mostly focused on plant–insect interaction, which is strongly influenced by biochemical compounds in plants [40,41,42]. However, the information about the effect of defoliators, including *L. monacha* and their control method on biologically active compounds in conifer needles is limited [43]. To fill the knowledge gap, we evaluated changes in the chemical structure, including variations in the content of polyphenols, flavonoids, photosynthetic pigments, lipid peroxidation, and soluble sugars in *P. sylvestris* needles following mass *L. monacha* outbreaks. In addition to the changes in the biochemical compounds induced by the expansion of the defoliator, the results of our study also revealed the potential effect of biological treatment with Foray 76B on these defensive compounds in pine needles.

Studies by other scientists have shown that secondary compounds protect trees against the hazardous effects of herbivorous insects [38,44]. The concentrations of secondary metabolites, such as terpenes and polyphenols, which exert a significant influence on the feeding performance of phytophagous insects [45], increase following insect damage in woody plants [46,47]. In response to stressful stimuli, plants exhibit a marked increment in the accumulation of phenolic compounds as an essential adaptation mechanism for tree survival [47,48]. Our study results indicated that in response to mass *L. monacha* outbreaks, the TPC in the needles of *P. sylvestris* trees also increased and was determined higher in the following year after the outbreaks. On the contrary, the TFC, which is one of the most abundant phenolic groups, was determined by lower concentrations in the needles from damaged plots. These trends of increased synthesis of polyphenols and decreased of flavonoids after the outbreaks confirm the results from other studies that the TPC does not correlate with the TFC, and changes in the concentration of polyphenols are mainly influenced by other their groups, i.e., phenolic acids, stilbenes or lignans [49]. Higher concentrations of the TFC in undamaged/healthy *P. sylvestris* forests indicate their importance in tree resistance against defoliating insects [50]. Even low concentrations of numerous flavonoids increase plant immune system [51] and might be a potential indicator of a higher forest stability and resistance to defoliators [50,52]. The results from our study showed that although treatment with Foray 76B had a temporary increment effect on the TFC, the changes did not remain in the following year after the outbreaks, leading to potential repeated damage by insect pests and pathogens in the future.

According to other studies, biotic disruptions, exemplified by the appearance of insect herbivore outbreaks or pathogen infestations, induce physiological stress in plants and can increase the production of photosynthetic pigments [51,53,54]. Photosynthesis-related pigments are characterized by their ability to protect the photosystems from photo radiation related to oxidative stress [55]. Our study revealed that in damaged by *L. monacha* outbreaks plots, the deteriorations of the concentrations of Chl a and Chl b were determined, with no effect on the concentration of Caro in *P. sylvestris* needles in the year when the outbreaks were recorded. Reduced levels of these chlorophylls observed in the needle samples of damaged pine plots potentially signify persistent biotic stress and a deteriorating state of the damaged trees. It is acknowledged that insect herbivory adversely affects the photosynthesis process through the regulation of gene expression associated with photosynthesis [56]. Among the methods for rapidly detecting plant stress, the fluorescence imaging of Chl a exhibits significant potential, as it enables the assessment of plant physiological status long before visible symptoms manifest [56]. Meanwhile, the concentrations of these photosynthetic pigments increased in the needles of damaged trees in the following year, which is important for faster forest stand recovery after such outbreaks [56,57]. The results of our study also revealed that the concentrations of Chl a, Chl b, and Caro increased in the needles of *P. sylvestris* in Foray 76B treated areas in the following year after the treatment, which might be influenced by *Bacillus thuringiensis* subspecies *kurstaki* Strain (Btk) (Foray 76B main component) [58,59]. While interactions between various *B. thuringiensis* strains and plants have been subject to limited investigation, some studies indicate that *B. thuringiensis* can be used as a plant growth-promoting agent by excreting phytohormones, such as auxins and siderophore precursors [58,59]. Although the findings from other studies indicate the potential ability of *B. thuringiensis* to increase the content of chlorophylls in plants [60,61,62], studies of the effect of coniferous forest treatment with Btk on tree response to biotic stress through their synthesis of chlorophylls in needles are lacking.

As the primary light-harvesting pigments, chlorophylls absorb light energy, converting it into chemical energy that is needed for sugar production [63,64]. Therefore, an increment in chlorophyll content in plants can lead to an increase in glucose and other soluble sugar production [65]. Sugars play a vital role in plant defense responses against various stressors by regulating signaling molecules related to plant immunity [66]. The results from our study showed that otherwise than chlorophylls, the concentration of TSS increased in the needles from damage by *L. monacha* outbreaks plots in the year when the outbreaks were recorded. However, the outbreaks did not influence the changes in the concentration of TSS in the following year after the outbreaks. The treatment with Foray 76B did not change the concentration of the TSS, in contrast to chlorophylls, which indicates that there is a complex relationship between the accumulation of chlorophylls and TSS that depends on various factors, such as the intensity and duration of stress or the individual characteristics of plant species [67].

In addition, biotic and abiotic plant stressors lead to excessive production of reactive oxygen species (ROS) within the plant system, culminating in the impairment of lipid structures, which induces the oxidative stress [68]. Particularly, malondialdehyde (MDA) is a biomarker of oxidative stress and lipid peroxidation [69]. Previous studies revealed that the levels of MDA in plants can either positively [41] or negatively [70] correlate with TPC after stress. The results of our study showed that *L. monacha* outbreaks influenced a decrease in MDA levels in *P. sylvestris* needles, especially in the following year after the outbreaks. It may be based on the ability of polyphenols to hinder lipid peroxidation and consequently maintain consistent levels of MDA [71]. When plants are exposed to stress conditions, including stressors such as insect outbreaks, the increased TPC can help to mitigate the damage caused by oxidative stress [72]. Our study revealed that treatment with Foray 76B did not affect the changes of MDA level in *P. sylvestris* needles in both years, which was related to no differences in TPC as well.

## 4. Materials and Methods

### 4.1. Study Sites and Sampling

**Figure 9:**
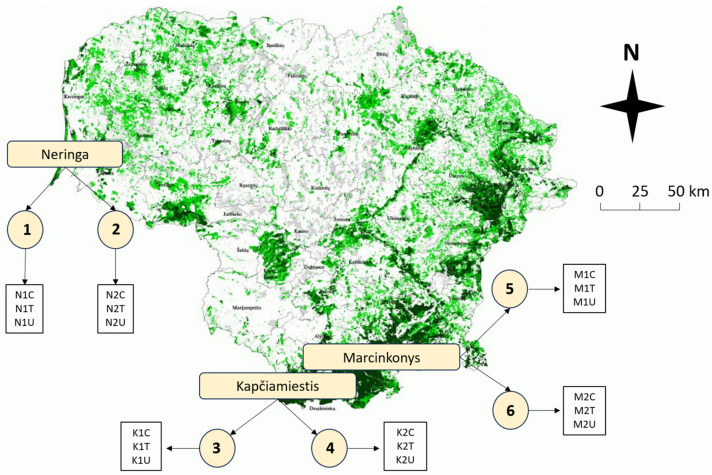
Map of Lithuania showing the distribution of *P. sylvestris* forest stands (green color) and their abundance in different areas. Circles (1–6) represent different forest stands within three plots (Control (C), Treated (T), Untreated (U)) at each forest stand. Descriptions of forest stand characteristics are presented in Table 3.

The study included a total of eighteen 50–120-year-old *P. sylvestris* plots in three different locations (Neringa, Kapčiamiestis, and Marcinkonys) in Lithuania (Figure 9). Two forest stands (codes: 1–2; 3–4; 5–6) were selected in each location, based on the forest inventory data, where *L. monacha* outbreaks were recorded and treatment with Foray 76B was applied (Figure 9, Table 3). Each forest stand represents three different plots: (1) visually healthy (C—Control); (2) damaged by *L. monacha* outbreaks and untreated (U—Damaged/Untreated); (3) damaged by *L. monacha* outbreaks and treated with Foray 76B (aerial spraying with the bioinsecticide (*Bacillus thuringiensis* subspecies *kurstaki* Strain *ABTS-351* (Btk)) was conducted in 2020 under the support of the State Forest Service) (T—Damaged/Treated). The variations in biochemical compounds in pine needles between Control plots and Damaged/Untreated plots were analyzed to assess the impact of *L. monacha* outbreaks. To evaluate the effect of the treatment with Foray 76B, the differences in biochemical compounds between Damaged/Treated and Damaged/Untreated plots were compared. Treated and untreated plots were selected with similar levels (30–60%) of tree defoliation. All the plots were selected with similar soil [73] and vegetation type [74], and all of them were characterized by normal humidity (N), very poor (a) or poor (b) fertility, light soil texture (l) and either *cladoniosum* (cl) or *vaccinio-myrtilliosum* (vm) vegetation type (Table 3). The sampling was performed two times, in October 2020 (the year when mass *L. monacha* outbreaks were recorded and treatment with Foray 76B was applied) and October 2021 (the year following the outbreaks and the treatment). At each of the eighteen plots (*n* = 18), five random order *P. sylvestris* trees were selected. From each tree, five branches with needles from 15 m above the ground and growing out from the main stem were cut with a telescopic pruner.

### 4.2. Extract Preparation

**Figure 10:**
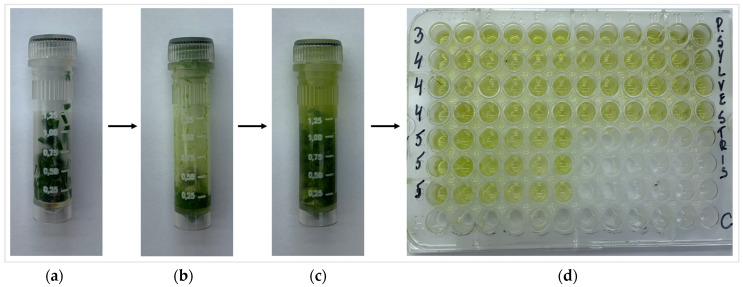
Preparation of *P. sylvestris* needle: (**a**) fresh needle sample in plastic tube; (**b**) needle sample after homogenization; (**c**) the homogenized needle sample with ethanol; (**d**) 96-well microplate with needle extracts.

The estimation of total phenolic content (TPC), total flavonoid content (TFC), chlorophyll a (Chl a), chlorophyll b (Chl b), carotenoids (Caro), level of lipid peroxidation (MDA), and total soluble sugars (TSS) was performed according the methodology of Čėsnienė et al., 2023 [47]. The weight of 0.1 g of fresh *P. sylvestris* needle samples were put in 2 mL plastic tubes (Sarstedt AG & Co. KG, Nümbrecht, Germany) (Figure 10) and homogenized using a Precellys homogenizer (Bertin Technologies, Montigny-le-Bretonneux, France) (5500 rpm, 30 s × 2). Then, 80% EtOH (2 mL) was added and homogenized again with the parameters of 4000 rpm for 30 s. After the homogenization, the tubes were centrifuged for 30 min, 21,910× *g*, +4 °C using a Hettich Universal 32R centrifuge (Andreas Hettich GmbH & Co. KG, Tuttlingen, Germany). Then, the tissue extracts were transferred to the 96-well microplates and mixed with different mixtures based on the analyzed biologically active compounds. Synergy HT Multi-Mode Microplate Reader (BioTek Instruments, Inc., Bad Friedrichshall, Germany) was used for the assessments.

### 4.3. Identification of Total Phenolic Content (TPC)

TPC was determined by using *Folin–Ciocalteu* reagent, according to Čėsnienė et al. (2023) [51] methodology. Analysis was performed in 96-well microplates; the reaction mixture consisted of *Folin–Ciocalteu reagent* (1:10) (VWR International GmbH, Vienna, Austria) and Na_2_CO_3_ (10%) (Firma Chempur, Piekary Śląskie, Poland). When 10 µL extract was mixed with 90 µL reaction mixture, samples were incubated at room temperature in the dark for 1 h. After incubation, the TPC was measured at a wavelength of 725 nm. Gallic acid (>98%, Carl Roth GmbH+Co. KG, Karlsruhe, Germany) was used as a standard and TPC was expressed as micrograms of gallic acid equivalents in one gram of fresh weight (mg GA/g FW). The calculation is shown in Formula (1). The calibration curve was: y = 0.093x + 0.0065 (R^2^ = 0.994).

Content (mg GA/g FW) = (C × V)/M,(1)

where C is the concentration obtained from the calibration curve (µg/mL); V—volume of the extract (mL); M—weight of the fresh biomass extracted (g).

### 4.4. Identification of Total Flavonoids Content (TFC)

TFC was detected based on the formation of a flavonoid–Al(III) complex [51]. Analysis of TFC was performed in 96-well micro plates; and the reaction mixture consisted of absolute EtOH (Merck KGaA, Darmstadt, Germany), AlCl_3_ (10%, *w*/*v*) (99% purity) (Alfa Aesar GmbH & Co KG, Karlsruhe, Germany), of C_2_H_3_KO_2_ (1M, 99% purity) (Sigma-Aldrich, Darmstadt, Germany) and dH_2_O. When 20 µL extract was mixed with 200 µL reaction mixture, samples were incubated at room temperature in the dark for 30 min. After incubation, the TPC was measured at a wavelength of 415 nm. Quercetin (>98%, Cayman Chemical Company, Ann Arbor, MI, USA) was used as a standard and TFC was expressed as micrograms of quercetin equivalent in one gram of fresh weight (mg QA/g FW). The calculation formula is shown in Formula (2). The calibration curve was y = 0.0366x + 0.0122 (R^2^ = 0.995).

Content (mg QA/g FW) = (C × V)/M(2)

where C is the concentration obtained from the calibration curve (µg/mL); V—volume of the extract (mL); M—weight of raw biomass extracted (g).

### 4.5. Quantification of Photosynthetic Pigments

Due to minimizing chlorophyll and carotenoid degradation, extracts were covered from light. The absorption of chlorophyll a (Chl a) chlorophyll b (Chl b) and carotenoids (Caro) were measured at wavelengths of 664 nm, 648 nm, and 470 nm, respectively. The concentrations of photosynthetic pigments were evaluated following the formulas produced by Beniušytė et al. (2023) [53]:

C(Chl a) = (13.36 × A_664_) − (5.19 × A_648_), (3)

C(Chl b) = (27.43 × A_648_) − (8.12 × A_664_), (4)

C(Caro) = (1000 × A_471_ − 2.13 × C(Chl a) − 97.64 × C(Chl b))/209 (5)

where A_absorbtion_ is absorption of the extract at the respective wavelength (664 nm, 648 nm, and 470 nm); C(Chl a), C(Chl b) and C(Caro) are concentrations of alpha and beta chlorophyll and carotenoids in the analyzing extract (μg/mL).

Total photosynthetic pigment concentration in a gram of fresh needle biomass is calculated according to formula:

Content (µg/g) = (C × V × W)/M,(6)

where C—concentration of Chl a, Chl b or Caro in the extract (μg/mL); V—volume of crude extract (mL); W—dilution of crude extract (units); M—weight of extracted biomass (g).

### 4.6. Identification of Levels of Lipid Peroxidation (MDA)

Levels of lipid peroxidation were determined by measuring the levels of malondialdehyde (MDA). The level of MDA in *P. sylvestris* needle tissue was evaluated using modified method, using thiobarbituric acid (TBA) [75,76,77]. Analysis was performed in 96-well micro plate. The reaction mixture consisted of 20% TCA (Molar Chemicals Kft, Halásztelek, Hungary) with 0.5% (*w*/*v*) TBA buffer (Alfa Aesar, Ward Hill, MA, USA). The volume of 50 µL needle extract was mixed with 130 µL reaction mixture. Then, samples were incubated in the heater (at 95 °C) for 30 min. (Agro-LAB Heating oven TCF 200, Venice, Italy). After the incubation, the reaction was stopped by quickly placing samples into the ice tube for cooling. Levels of MDA were measured at wavelengths of 440 nm, 532 nm, and 600 nm. The levels of MDA were expressed as nmol MDA per gram of crude tissue based on dilution and sample weight. MDA concentration was calculated using the following Formula (7).

MDA (nmol MDA/g FW) = 6.45 × (A_532_ − A_600_) − (0.56 × A_440_), (7)

where A_absorption_ is absorption of the extract at the respective wavelength (532, 600 or 440).

### 4.7. Evaluation of Total Soluble Sugars (TSS)

The concentration of TSS was determined according to the Leyva et al. (2008) method [78]. Analysis was performed in heating 96-microplates plate; and the reaction mixture consisted of anthrone reagent (0.1%, diluted in concentrated sulfuric acid) (Carl Roth GmbH+Co. KG, Karlsruhe, Germany). When 10 µL extract was mixed with 90 µL reaction mixture, samples were covered with foil and incubated in the heater (at 90 °C) for 1 h (Agro-LAB termoastating TFC 200, Venice, Italy). After incubation, samples were cooled, and the concentration of TSS was measured at wavelengths of 620 nm. Glucose (Firma Chempur, Piekary Śląskie, Poland) is used to create the calibration curve and the amount of TSS was expressed as glucose equivalents (mg) per gram of raw tissue based on dilution and sample weight. The calibration curve was y = 0.002x + 0.234 (R^2^ = 0.999).

The final quantity of TSS was calculated using the Formula (8):

TSS (mg/g FW) = C/V/M,(8)

where C is the concentration obtained from the calibration curve (µg/mL); V—volume of the extract (mL); M—weight of raw biomass extracted (g).

### 4.8. Data Analysis

For the assessment, the mean numbers of each analyzing variable (control, treated, and untreated) from all locations together (Neringa + Kapčiamiestis + Marcinokonys) were calculated (*n* = 18; 3 locations × 2 forest stands × 3 plots). For the assessment of biologically active compounds 3 biological × 3 technical replicates were used. All statistical data was performed using R (Version 4.2.1) with RStudio (Version 1.1.456) programming language and Microsoft Excel 2010. The column charts with error bars represent the mean number of values with the standard error (SE), indicated as mean ± SE. The boxplots were performed for the assessment of median values, their spread, and the presence of outliers. For estimation of statistical differences between analyzed groups, ANOVA was tested, followed by a Tukey HSD (honestly significant differences). The statistically significant difference between the two groups, represented by different lowercase letters, was considered when the results of the Tukey HSD and ANOVA were less than 0.05. The Pearson correlation coefficient was calculated with RStudio using the corrr library, while the visualization of the correlation matrix was performed using ggcorrplot function from ggplot2 package. For the performance and visualization of the distribution of biochemical compounds, principal component analysis (PCA) was performed using ggplot2, vegan, FactoMineR, FactoExtra, and ggrepel libraries.

## 5. Conclusions

The conducted study illustrates the significant effect of *L. monacha* outbreaks on the content of various biochemical compounds in the needles of *P. sylvestris* trees. The increased TPC after the outbreaks was negatively correlated with decreased TFC which was determined in both 2020 and the following year after the outbreaks. Higher TPC in damaged plots indicates that weakened trees are still in the defense and recovery process in the following year after the outbreaks. The increased TPC was also related to lower levels of MDA, which particularly shows the importance of polyphenols to hinder concentrations of lipid peroxidation and mitigate tree damage caused by oxidative stress during biotic stress. The increased synthesis of chlorophylls and carotenoids in damaged plots in the following year after the outbreaks is relevant for biochemical processes, such as glucose production or photo-protectors, which in turn lead to faster tree recovery. Furthermore, our study assessed the stimulating effect of the treatment with biological insecticide Foray 76B on chlorophylls and carotenoids in the following year after the outbreaks. The treatment did not influence other changes in assessed biochemical compounds. Although the obtained results enhance our understanding of the response of *P. sylvestris* to *L. monacha* outbreaks and treatment with Foray 76B through the tested compounds in pine needles, further studies are needed to demonstrate the longer-term processes in tree needles that influence pine recovery after such biotic stress.

## Figures and Tables

**Table 1:** Standard deviation, proportion of variance, and cumulative proportion explained by principal components (PC1–PC7) through analysis of PCA from the data of 2020 and 2021.

Importance of Components (2020)	PC1	PC2	PC3	PC4	PC5	PC6	PC7
Standard deviation	1.739	1.172	0.911	0.826	0.776	0.621	0.247
Proportion of Variance	0.435	0.197	0.119	0.098	0.087	0.055	0.009
Cumulative Proportion	0.435	0.632	0.751	0.849	0.936	0.991	1.000
**Importance of Components (2021)**	**PC1**	**PC2**	**PC3**	**PC4**	**PC5**	**PC6**	**PC7**
Standard deviation	1.448	1.311	0.954	0.933	0.840	0.702	0.402
Proportion of Variance	0.301	0.247	0.131	0.125	0.101	0.071	0.023
Cumulative Proportion	0.301	0.548	0.679	0.805	0.906	0.977	1.000

**Table 2:** Individual proportion of the traits to the 1st and 2nd components from the data of 2020 and 2021.

Trait	2020		2021	
	1st Component	2nd Component	1st Component	2nd Component
TPC	0.06144	0.49082	0.28465	0.01474
TFC	0.16538	−0.48788	0.13065	0.67010
Chl a	−0.64602	−0.07751	0.53737	−0.13954
Chl b	−0.59924	−0.14574	0.50337	−0.08715
Caro	−0.39843	0.40333	0.47682	−0.07655
MDA	−0.03230	−0.47597	0.03444	0.71146
TSS	0.18071	0.32341	0.36223	0.10796

**Table 3:** Characteristics of forest stands, representing control, treated, and untreated *P. sylvestris* plots. Data was obtained by permission from the State Forest Cadaster as of 2021.

Location	Forest Stand Code	Plot Code	Group	Geographical Position		Stand Characteristics						
				N	E	Age (y)	Mean Height (m)	Mean Diameter (cm)	Stocking Level	Forest Site Type *	Forest Vegetation Type **	Tree Species Composition, % ***
Neringa	1	N1C	Control	55.62489	21.112491	50	17.1	20.5	0.89	Nal	cl	100% *P*
		N1T	Damaged/Treated	55.69654	21.106127	120	16.5	23.0	1.00	Nal	cl	100% *P*
		N1U	Damaged/Untreated	55.67744	21.106809	65	16.7	18.0	0.84	Nal	cl	100% *P*
	2	N2C	Control	55.53548	21.10357	92	19.2	30.4	0.67	Nal	cl	100% *P*
		N2T	Damaged/Treated	55.33166	21.041377	60	18.1	19.0	1.04	Nal	cl	100% *P*
		N2U	Damaged/Untreated	55.40839	21.078366	110	11.5	13.0	1.16	Nal	cl	100% *P*
Kapčiamiestis	3	K1C	Control	54.01370	23.519638	94	27.1	31.8	0.92	Nbl	vm	100% *P*
		K1T	Damaged/Treated	54.04471	23.537946	89	29.3	34.0	0.92	Nbl	vm	100% *P*
		K1U	Damaged/Untreated	54.03606	23.535732	74	23.6	28.7	0.90	Nbl	vm	100% *P*
	4	K2C	Control	53.97177	23.507236	99	28.0	33.8	0.70	Nbl	vm	100% *P*
		K2T	Damaged/Treated	54.01868	23.525614	89	25.1	29.9	0.80	Nbl	vm	100% *P*
		K2U	Damaged/Untreated	54.00465	23.501548	76	28.2	31.3	0.92	Nbl	vm	100% *P*
Marcinkonys	5	M1C	Control	54.09489	24.45175	90	27.0	32.0	0.81	Nbl	vm	100% *P*
		M1T	Damaged/Treated	54.10660	24.433008	98	28.6	35.4	0.80	Nbl	vm	100% *P*
		M1U	Damaged/Untreated	54.12597	24.454743	78	25.2	25.6	0.81	Nbl	vm	100% *P*
	6	M2C	Control	54.05331	24.443881	85	26.1	30.2	0.74	Nbl	vm	100% *P*
		M2T	Damaged/Treated	54.03104	24.435073	125	25.3	25.7	0.71	Nbl	vm	100% *P*
		M2U	Damaged/Untreated	54.03598	24.424077	83	26.8	29.6	0.81	Nal	cl	100% *P*

* N: Normal humidity, a: very poor fertility, b: poor fertility, l: light soil texture. ** cl: *cladoniosum*, vm: *vaccinio-myrtilliosum* [73]. *** *P: Pinus sylvestris*. In each stand, tree species composition is based on the volume.

## Data Availability

Main data of this study are provided in an article. Detailed calculations are not provided due to extremely big amount of the data. The whole package of the data is available upon request.

## Abbreviations

Caro	carotenoids
Chl a	chlorophyll a
Chl b	chlorophyll b
Btk	*Bacillus thuringiensis* subspecies *kurstaki* Strain ABTS-351
MDA	malondialdehyde
ROS	reactive oxygen species
PSMs	plants secondary metabolites
TFC	total flavonoid content
TPC	total phenolic content
TSS	total soluble sugars
